# Sildenafil ameliorates biomarkers of genotoxicity in an experimental model of spontaneous atherosclerosis

**DOI:** 10.1186/1476-511X-12-128

**Published:** 2013-08-28

**Authors:** Bianca P Rodrigues, Bianca P Campagnaro, Camille M Balarini, Thiago M C Pereira, Silvana S Meyrelles, Elisardo C Vasquez

**Affiliations:** 1 Laboratory of Translational Physiology, Health Sciences Center, Federal University of Espirito Santo (UFES), Vitoria, Brazil; 2 Pharmaceutical Sciences Graduate Program, University of Vila Velha (UVV), Vila Velha, ES, Brazil; 3 Federal Institute of Education, Science and Technology (IFES), Vila Velha, ES, Brazil; 4 Emescam School of Health Sciences, Vitoria, Brazil

## Abstract

**Background:**

It is well known that enhanced production of reactive oxygen species (ROS) leads to oxidative stress observed in atherosclerosis and that ROS can also cause damage in cellular macromolecules, including DNA. Considering previous report that sildenafil, an inhibitor of phosphodiesterase 5 (PDE5), has antioxidant effects, in the present study we evaluated the effect of this drug on genotoxicity of blood mononuclear cells (MNC) and liver cells from atherosclerotic apolipoprotein E knockout mice (apoE^-/-^).

**Methods:**

ROS production in MNC was evaluated by flow cytometry with the fluorescent dye dihydroethidium (DHE), a method that has been used to quantify the production of superoxide anion, and DNA damage was evaluated in both MNC and liver cells using the alkaline comet assay. Sildenafil-administered apoE^-/-^ mice were compared with strain-matched mice administered with vehicle and with C57BL/6 wild-type (WT) mice.

**Results:**

MNC from apoE^-/-^ vehicle exhibited a 2-fold increase in production of superoxide anion in comparison with WT. In contrast, sildenafil-administered apoE^-/-^ mice showed superoxide anion levels similar to those observed in WT mice. Similarly, MNC and liver cells from apoE^-/-^ vehicle mice showed a 4-fold and 2-fold augmented DNA fragmentation compared with WT, respectively, and sildenafil-administered apoE^-/-^ mice exhibited minimal DNA damage in those cells similar to WT mice.

**Conclusions:**

ApoE^-/-^ mice chronically administered with sildenafil exhibited reduced levels of superoxide anion in MNC and less DNA fragmentation in MNC and liver cells, which are biomarkers of genotoxicity. Therefore, sildenafil may offer a new perspective to the use of PDE5 inhibitors to protect against DNA damage, in cells involved in the inflammatory and dyslipidemic processes that accompany atherosclerosis.

## Background

Hypercholesterolemia and atherosclerosis results from metabolic disorders, enhanced oxidative stress and inflammation [1-3]. Experimentally, the apolipoprotein E knockout mouse (apoE^-/-^) has been widely used in studies aiming to better understand this disease and to propose new treatment approaches. In this model, the atherosclerotic process increases continuously and the progression of lesions is accelerated under Western-type diet [4-6].

Experimental and clinical evidences support the hypothesis that lipid-oxidation products, obtained endogenously or ingested with food, increases incidence of atherosclerosis [7-10] and even tumor frequency [8,9]. These effects are justified by genotoxicity in various locals, including blood cells and hepatocytes [7]. Furthermore, it has been suggested that the excessive generation of reactive oxygen species (ROS), leading to the oxidative stress play an important role in the induction of DNA damage [6,11].

Oxidative stress is the result of an imbalance between the production of oxidant species and antioxidant defences, with predominance of ROS [6,12]. High levels of ROS are important mediators of damage in cell components such as carbohydrates, lipids, proteins and nucleic acids [13]. Oxidative damage to DNA can occur in different ways, causing oxidation of specific bases or strand breaks, leading to genomic instability and permanent changes in the genetic material (genotoxicity) [14]. Thus, in conditions of increased oxidative stress, as observed in atherosclerosis, antioxidant alternative strategies could be convenient to reduce oxidative stress and to prevent the genetic material damage. Experimentally, the comet assay evaluates DNA damage, which is a biomarker of genotoxicity, in individual cells through the measurement of DNA migration in gel electrophoresis [15]. Although it has been usually performed in blood cells, which are easily obtained to demonstrate systemic genotoxic damage, other tissues can also be used, as the effects of genotoxicity are tissue-specific [16,17]. Liver is also considered a target organ for genotoxicity research, specifically in atherosclerosis, as this is the main organ of lipid metabolism [16,17].

Recently our laboratory showed that sildenafil, a phosphodiesterase 5 (PDE5) inhibitor which has been widely used for erectile dysfunction and pulmonary hypertension treatment [18,19], restores endothelial function in apoE^-/-^ mice [20]. Considering experimental evidence that this drug can prevent oxidative stress induction and lipid peroxidation [19,20], sildenafil could be a promising pharmacological alternative to prevent ROS-induced DNA damage in atherosclerosis. Therefore, the aim of the present study was to evaluate the effect of sildenafil on genotoxicity induced by oxidative stress of mononuclear cells (MNC) and liver cells of atherosclerotic apoE^-/-^ mice.

## Results

### Lipid profile

Table 1 summarizes average values of lipid profile in wild-type (WT), apoE^-/-^ vehicle and apoE^-/-^ sildenafil. As expected and consistent with classical and recent data [20-26], the apoE^-/-^ mice showed higher total plasma cholesterol (12-fold), low density lipoproteins (LDL, 5-fold), very low density lipoproteins plus intermediate density lipoproteins (VLDL + IDL, 56-fold) and triglycerides (5-fold) than the WT animals; the values of high density lipoproteins (HDL) were significantly decreased (2-fold) compared with WT animals. Treatment with sildenafil did not change this lipid profile in apoE^-/-^ mice.

**Table 1:** Plasma Lipid profile

**Parameters**	**Groups**		
	**Wild-type**	**apoE**^**-/- **^**vehicle**	**apoE**^**-/- **^**sildenafil**
Triglycerides (mg/dL)	52 ± 6.1	299 ± 47**	248 ± 52*
Total cholesterol (mg/dL)	100 ± 7.8	1229 ± 185**	1310 ± 222**
LDL (mg/dL)	34 ± 7.2	188 ± 49**	217 ± 48.6**
HDL (mg/dL)	49 ± 2.8	24 ± 8**	10 ± 0.9**
VLDL + IDL (mg/dL)	18 ± 5.5	1016 ± 195**	1083 ± 240**

Values are means ± SEM; 6-8 animals per group.

*p < 0.05 and **p < 0.01 vs. Wild-type group (ANOVA).

### Reactive oxygen species (ROS)

**Figure 1:**
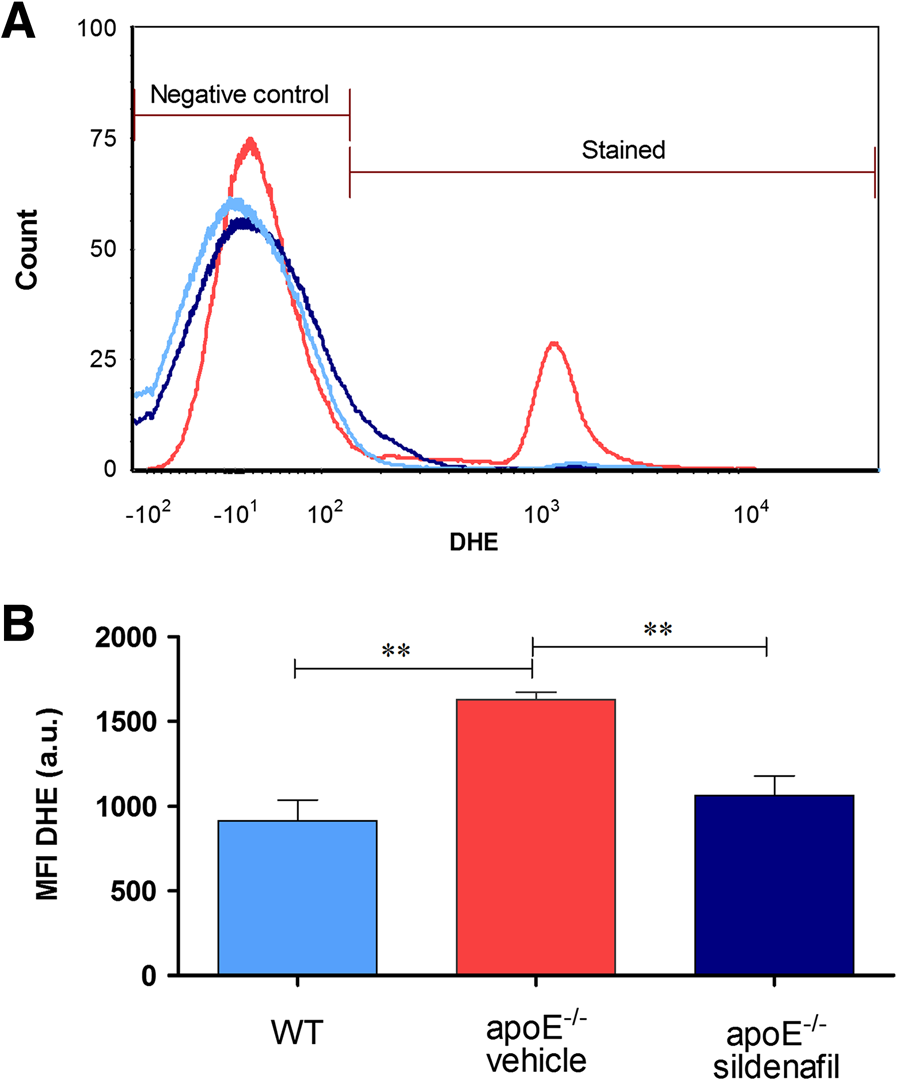
**Effect of sildenafil treatment on superoxide anion production in MNC of apoE**^**-/- **^**mice. (A)** Representative histogram from flow cytometric analysis using dihydroethidium (DHE) in control C57 wild-type (WT), apoE^-/-^ vehicle and apoE^-/-^ sildenafil; the log fluorescence (X axis) illustrates the intensity of fluorescence for the number of cells counted (note the higher scales in the apoE^-/-^ vehicle histogram). A remarkable increase in the level of superoxide anions was observed in apoE^-/-^ vehicle. Line colors correspond to bar colors below. **(B)** Bar graph showing mean fluorescence intensity (MFI, in a.u.). The values are presented as means ± SEM; 6 to 8 animals per group. **p < 0.01, ANOVA).

The term oxidative stress is often used to imply a condition in which cells are exposed to excessive levels of either molecular oxygen or chemical derivatives of oxygen [27]. Our laboratory has previously shown the applicability of the method of flow cytometry with dihidroethidium (DHE) to evaluate the production of superoxide anions in cells from the apoE^-/-^ mouse [11]. The presence of superoxide anions is indicated by the median fluorescence intensity (MFI, in a.u.). Typical histogram from flow cytometric analysis show a rightward-shift in the log of DHE fluorescence in apoE^-/-^ vehicle (Figure 1A) compared with WT, which contrasts with apoE^-/-^ sildenafil. As expected, we observed in Figure 1B a remarkable increase in the levels of superoxide anions in apoE^-/-^ vehicle mice (1629 ± 44 a.u., p < 0.01) compared with WT mice (915 ± 124 a.u.) and treatment with sildenafil was able to decrease these levels in apoE^-/-^ mice (1065 ± 115 a.u., p < 0.01).

### Analysis of DNA damage by comet assay

**Figure 2:**
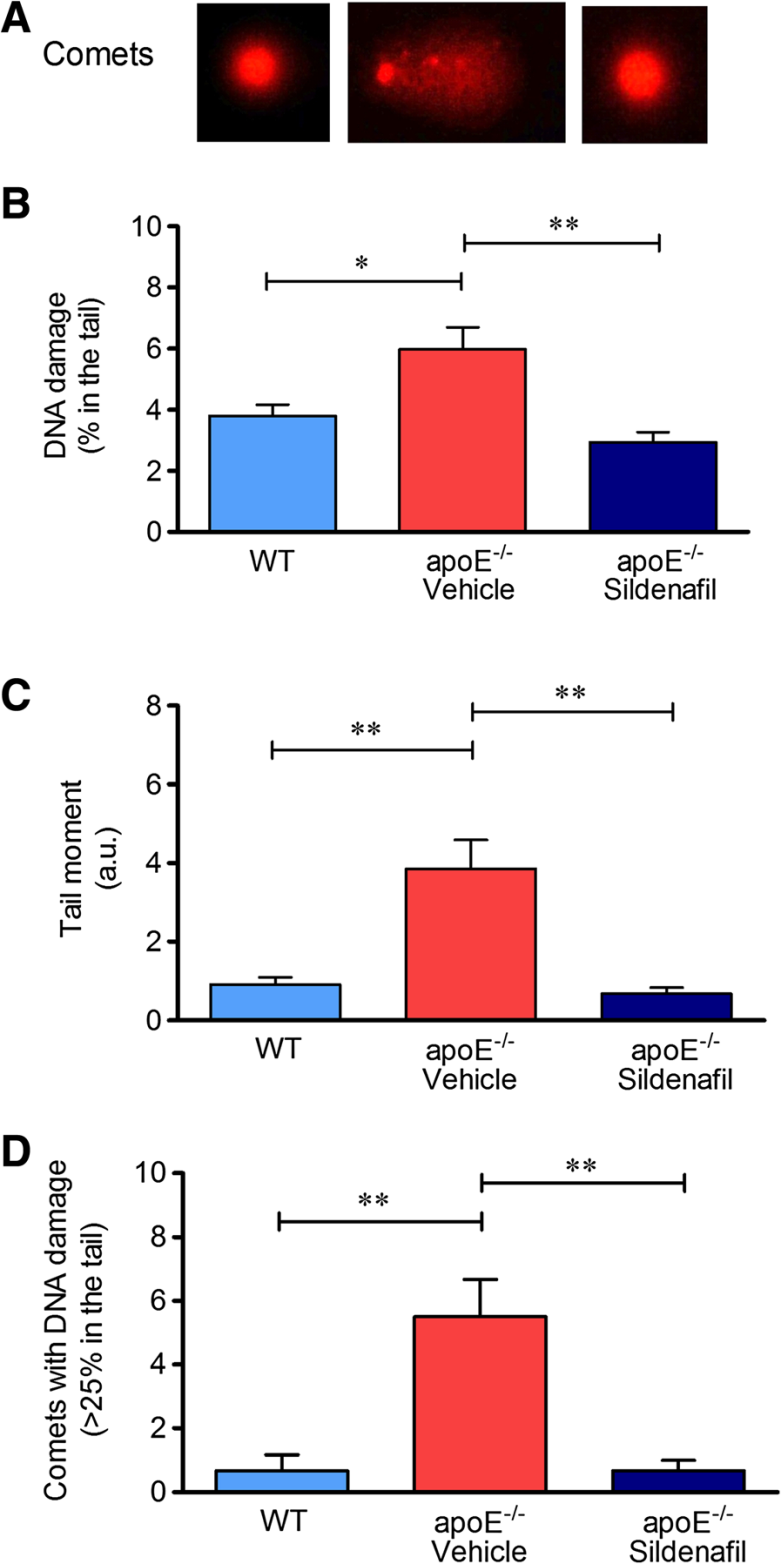
**Detection of DNA damage in MNC assessed by alkaline comet assay. (A)** Typical comets showing higher DNA fragmentation in apoE^-/-^ vehicle compared to control WT, which contrasts with apoE^-/-^ sildenafil; **(B)** Bar graph showing the mean of percentage of DNA in tail; **(C)** Bar graph showing the mean of tail moment (in a.u.); **(D)** Bar graph showing the mean of the number of comets with moderate-to-high damage (more than 25% of DNA in the tail). The values are presented as means ± SEM; 6 to 8 animals per group. *p < 0.05 and **p < 0.01, ANOVA).

**Figure 3:**
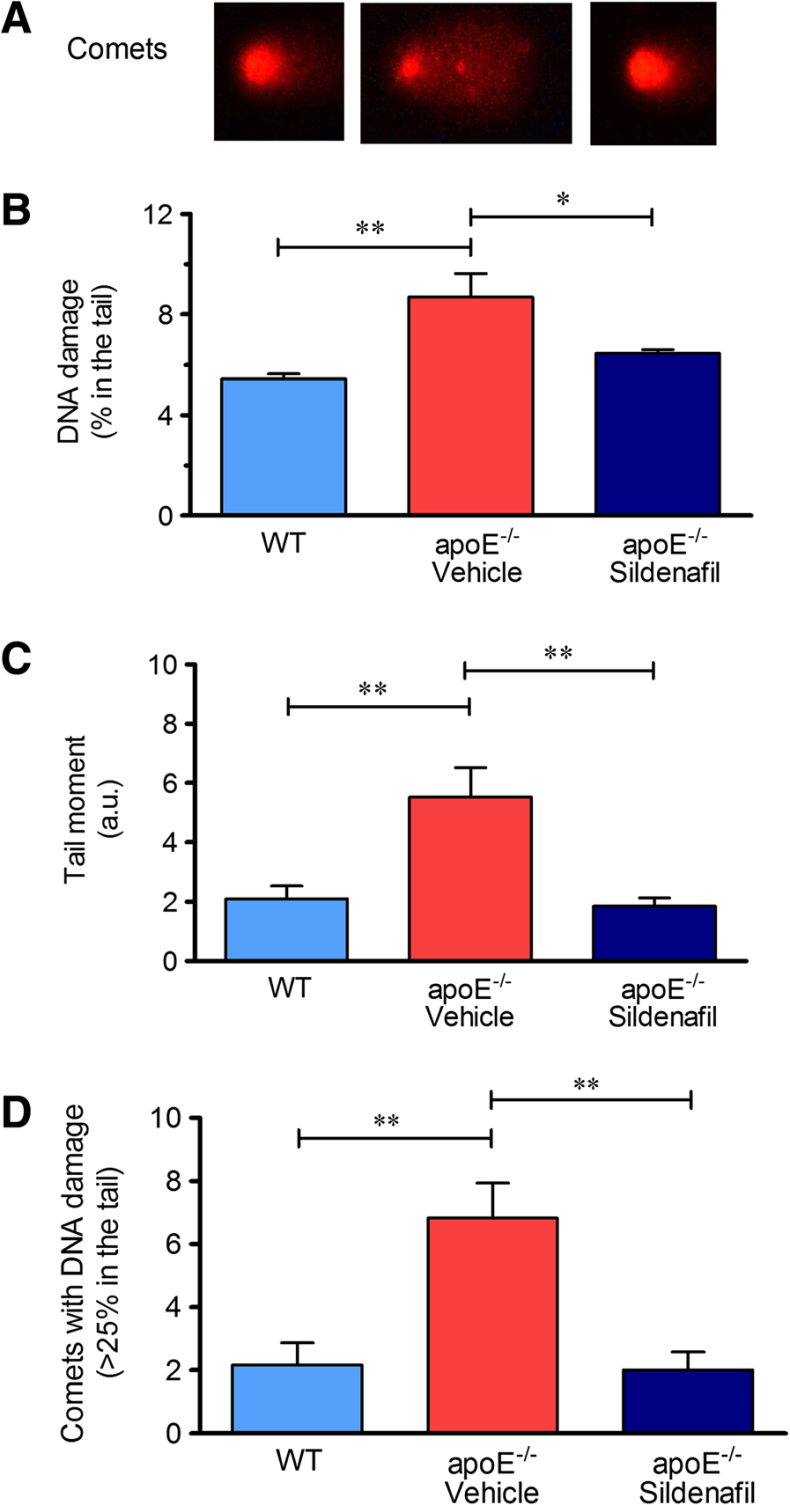
**Detection of DNA damage in liver cells assessed by alkaline comet assay. (A)** Typical comets showing higher DNA fragmentation in apoE^-/-^ vehicle compared to control WT, which contrasts with apoE^-/-^ sildenafil; **(B)** Bar graph showing the mean of percentage of DNA in tail; **(C)** Bar graph showing the mean of tail moment (in a.u.); **(D)** Bar graph showing the mean of the number of comets with moderate-to-high damage (more than 25% of DNA in the tail). The values are presented as means ± SEM; 6 to 8 animals per group. *p < 0.05 and **p < 0.01, ANOVA).

The genotoxicity analysis was performed by the alkaline comet assay which measures single and double strand breaks as well as lesions that are converted to strand break by the alkaline pre-treatment (i.e., alkaline labile sites) [5]. In the comet assay, the percentage of DNA in the tail represents the number of fragments that migrated during electrophoresis. Figures 2A and 3A represent typical comets of MNC and liver cells (respectively), showing higher DNA fragmentation in apoE^-/-^ vehicle compared to control WT, and that it was reduced in apoE^-/-^ sildenafil. Figure 2B shows the results of the average percent of DNA in the tail of MNC (WT: 3.8 ± 0.4% vs. apoE^-/-^ vehicle: 6.0 ± 0.7%, p < 0.05 vs. apoE^-/-^ sildenafil: 2.9 ± 0.3%, p < 0.01). Similarly, Figure 3B shows the results of the average percent of DNA in the tail of liver cells (WT: 5.5 ± 0.2% vs. apoE^-/-^ vehicle: 9.0 ± 1.2%, p < 0.01 vs. apoE^-/-^ sildenafil: 6.4 ± 0.2%, p < 0.05). As shown, apoE^-/-^ vehicle group showed higher DNA damage when compared to WT and it was demonstrated that apoE^-/-^ mice administered with sildenafil exhibit minimal DNA damage comparable with those observed in WT control mice.

Another parameter analyzed was the comet tail moment, an index of both the migration of the genetic material and the relative amount of DNA in the tail [28]. This analysis revealed a decrease of DNA fragmentation in animals administered with sildenafil in both MNC (Figure 2C: WT: 0.9 ± 0.2 a.u. vs. apoE^-/-^ vehicle: 3.9 ± 0.7 a.u., p < 0.01 vs. apoE^-/-^ sildenafil: 0.7 ± 0.2 a.u., p < 0.01) and liver cells (Figure 3C: WT: 2.1 ± 0.4 a.u. vs. apoE^-/-^ vehicle: 5.5 ± 1.0 a.u., p < 0.05 vs. apoE^-/-^ sildenafil: 1.9 ± 0.3 a.u., p < 0.01). In addition, we also quantified the comets with moderate-to-high damage based on the cutoff of more than 25% of DNA on the tail. This parameter is summarized in the Figure 2D, which shows that the number of comets with more than 25% of DNA on the tail in MNC were significantly higher in apoE^-/-^ vehicle than in WT control mice (5.5 ± 1.2 **vs.** 0.8 ± 0.5, p < 0.01) and normalized in apoE^-/-^ sildenafil mice (0.7 ± 0.3, p < 0.01). Similar results were observed in liver cells, as shown in Figure 3D (WT: 2.2 ± 0.7 vs. apoE^-/-^ vehicle: 6.8 ± 1.1, p < 0.01 vs. apoE^-/-^ sildenafil: 2.0 ± 0.6, p < 0.01). Taken together, these results demonstrate that chronic administration of sildenafil is able to reduce DNA fragmentation in MNC and liver cells of apoE^-/-^ mice.

## Discussion

Cumulative evidence suggests that DNA instability plays an important role in chronic degenerative diseases as atherosclerosis [29,30]. In this study, we reported for the first time that chronic inhibition of PDE5 with sildenafil can decrease genotoxicity in MNC and liver cells *in vivo*, in atherosclerotic apoE^-/-^ mouse model.

Interestingly, sildenafil did not change the plasma lipid profile in apoE^-/-^ mice, which is consistent with results previously reported by our laboratory [20] and by others [23]. Although Ronsein et al. [31] have proposed that hypercholesterolemia directly contributes to the DNA damage, which was also demonstrated in mice [11,17], rats [32] and rabbits [33], in the present study we show evidence that the amelioration in oxidative stress and DNA damage by sildenafil might be independent of hypercholesterolemia.

Elevated levels of ROS, which is a characteristic of a state of oxidative stress, has been reported in clinical and pre-clinical studies in many cardiovascular diseases, including diabetes, hypertension and atherosclerosis [3,11,24,34,35]. Although under physiological conditions ROS are continuously produced in most cells, the imbalance between ROS production and its degradation, can lead to genomic instability and, consequently, permanent changes in the genetic material, contributing to unfavourable processes, *e.g.* apoptosis, observed in different target tissues of the cardiovascular diseases [17,36].

Oxidative DNA damage can result from a variety of factors including radiation, toxins, chemicals and ROS, by products of normal metabolic processes [37]. It is well-known that DNA damage can occur in cells exposed to oxidative stress and the oxidative DNA damage has been estimated as 10^4^ hits per cell per day in humans; in this way, oxidative stress could be the main contributor to DNA damage in cardiovascular diseases [30,38,39].

Recently, we reported that sildenafil appears to involve an enhancement of the nitric oxide (NO) pathway along with a reduction in oxidative stress [20]. Moreover, it has been shown that increased intracellular levels of cGMP can inhibit NADPH oxidase expression/activity [20,40,41], increase enzyme activities of superoxide dismutase, catalase and glutathione peroxidase [19], thereby reducing superoxide anion bioavailability. The antioxidant properties of sildenafil [12] could constitute a key mechanism for the decrease of oxidative stress and DNA damage that we observed in MNC and liver cells. Another potential advantage of a therapeutic approach based on inhibition of PDE5 is that, unlike strategies that attempt to increase NO levels (e.g., substrates, co-factors, antioxidants) [42,43], this one avoids risks associated with NO, which in excess is a cytotoxic free radical which possess the characteristic of a “double-edged sword” [44]. Accordingly, it has been demonstrated that at high concentrations, NO leads to increases in the production of peroxynitrite and oxidative stress [45], with consequent DNA damage [46]. Finally, the beneficial pleiotropic effects of sildenafil could also be explained, by pharmacokinetic and pharmacodynamics characteristics: high volume of distribution [47] and ubiquity of the second-messenger target (cGMP) in cytoprotective signaling pathways [48] distributed in various tissues/cells expressing PDE5 such as MNC [49] and liver cells [50-52]. Our findings are in agreement with recent studies in many experimental models, which have demonstrated a protection of MNC [53] and prevention of liver injury by sildenafil through a direct contribution of the NO/cGMP mechanism [52,54,55].

The comet assay used for analyses of DNA is a well-established, simple, sensitive and extensively used tool to evaluate genotoxicity by comet tail parameters that are related to the amount of damage in nuclear DNA [56]. An advantage of this technique is the possibility of its use in different cell types when it is suggested that the genotoxicity is tissue specific [16,17]. Some authors have suggested that comet pictures might be associated with apoptotic cell nuclei, but they have limited this interpretation to highly damaged cells called “ghost cells” that are easily recognizable in situ on the slides [57,58]. Previous studies have shown that the apoptosis could influence the parameters of the comet tail, but not enough to distort the interpretation of the results [58]. Additionally, the present study supports the idea that the comet assay is a useful approach to assess the efficacy in the treatment on genotoxicity of MNC and liver cells, even in a short time interval (3 weeks).

The importance of evaluating MNC is based in the fact that atherosclerosis is an inflammatory disease [3,25] that causes an increase in recruitment of inflammatory leukocytes, mainly the MNC types. Likewise, the liver was focused due to its metabolic importance and to its high susceptibility to cumulative oxidized products of DNA [17]. Furthermore, Folkmann et al. [17] showed that dyslipidemic apoE^-/-^ mice suffer from hepatic oxidative stress/genotoxicity and this could be due to dysfunction of the lipid metabolism. The novelty of the present study is that it was possible to reduce DNA damage in MNC and liver cells of apoE^-/-^ mice by chronic inhibition of PDE5 with sildenafil, even under conditions of hypercholesterolemia, possibly by the same antioxidative mechanisms above commented. This finding supports the idea that sildenafil is a promising novel pharmacologic strategy to avoid tissue damage induced by oxidative stress as previously reported by us [20] and others [40,41,48], thus opening the way for translational studies about the protection of DNA in different clinical conditions.

### Study strength and limitations

A major strength of our study is the evaluation of the beneficial effects of sildenafil on genotoxicity in the apoE^-/-^ mouse, which exhibited a protective action against DNA damage in MNC and liver cells. However, this study has some limitations. Although it has been demonstrated that sildenafil administered for 3 weeks reduces oxidative stress in smoke induced erectile dysfunction in C57BL6 mice [59] and has been considered a novel therapeutic strategy to repair the endothelial dysfunction in apoE^-/-^ mice [20], we cannot predict whether such beneficial effects on genotoxicity and oxidative stress are long-lasting in atherosclerosis. Another limitation of the present study is that we did not include in our protocol a group of apoE^-/-^ under a regular chow.

## Conclusions

ApoE^-/-^ mice are characterized by a systemic oxidative stress, as demonstrated by MNC high levels of superoxide anion production that leads to DNA damage. In these animals, hepatic oxidative stress (in terms of DNA damage) is also substantial, when compared to control normocholesterolemic animals. The treatment with sildenafil was efficient to decrease the levels of superoxide anion in MNC and the DNA fragmentation in both MNC and liver cells in apoE^-/-^ mice. Thus, we propose that sildenafil may offer a new perspective to the use of PDE5 inhibitors to protect against DNA damage observed in atherosclerosis, independent of hypercholesterolemia.

## Methods

### Animals

Experiments were performed in male WT (C57BL6) and apoE^-/-^ mice obtained from the Laboratory of Transgenes in the Health Sciences Center at the Federal University of Espirito Santo, Brazil. Animals were housed in individual plastic cages with a controlled temperature (22-23°C) and humidity (60%) and were exposed to a 12:12-h light–dark cycle. All experimental procedures were performed in accordance with the guidelines for the care and handling of laboratory animals as recommended by the National Institutes of Health (NIH), and study protocols were previously approved by the Institutional Animal Care and Use Committee (CEUA-Emescam, Protocol 007/2010).

To accelerate and aggravate the spontaneous hyperlipidemia and atherosclerosis in apoE^-/-^ mice, 8-week-old animals were fed a Western-type diet (AIN93G modified diet, Rhoster, Brazil). Animals were distributed into three different groups: (a) apoE^−/−^ mice administered with the PDE5 inhibitor sildenafil (apoE^−/−^ sildenafil, Viagra®, 40 mg/kg/day, for 3 weeks, by oral gavage), (b) apoE^-/-^ mice administered with vehicle and (c) WT control mice. This dose of sildenafil has been previously used in apoE^-/-^ mice by Dussault et al. [23] and by our laboratory [20] in studies about endothelial dysfunction, based on the fact that this drug has reduced oral bioavailability by pre-systemic hepatic metabolism besides high clearance in mice [60]. All animals had *ad libitum* access to water and food during housing and treatment periods. For each protocol were used 6 to 10 animals per group.

### Samples

Animals were euthanized with sodium thiopental overdose (100 mg/kg, IP). A thoracic incision was performed for blood collection trough intra-cardiac puncture. Blood was immediately transferred to a tube containing EDTA. Peripheral blood mononuclear cells (MNC) were isolated by Histopaque® density gradient centrifugation, according to the manufacturer’s instructions. The samples were stored at -80°C until further analysis.

Liver cells enriched fractions from the mice liver of different groups were prepared as standardized in our laboratory based on previous studies [5,17]. The left lobe of the liver was grossly triturated with surgical scissors and incubated with an extraction solution containing proteinase K (Sigma-Aldrich, St. Louis, MO, USA) and collagenase type II (Gibco Life Technologies, São Paulo, SP, Brazil) to dissociate the cells. Then, the cell extract was filtered through a nylon screen (BD falcon 70 μm) to remove cell debris. After, the samples were washed twice in phosphate-buffered saline (PBS) to remove the enzymes. The samples were stored at -80°C until further analysis.

Before performing analyzes of ROS production and genotoxicity was carried out viability test through trypan blue exclusion test and both MNC and liver cells showed 80–90% viability.

### Measurement of lipid profile

The plasma of peripheral blood samples was used to measurement of lipid profile, total plasma cholesterol, HDL, LDL and triglycerides were determined using commercial colorimetric assay kits (Bioclin, Belo Horizonte, Brazil). VLDL and IDL were estimated by subtracting HDL and LDL from total serum cholesterol.

### Measurement of cytoplasmic reactive oxygen species by DHE

DHE was used for the flow cytometry detection of intracellular superoxide anion. DHE is freely permeable to cells and is rapidly oxidized, mostly by superoxide, to ethidium, which binds to DNA and amplifies red fluorescence signal. To estimate the content of superoxide anion in cell suspension, 10^6^ MNC were incubated with 20 μL of DHE (160 μM) for 30 min at 37°C in the dark to load the cells with the dyes [11,35]. For positive control, samples were treated for 5 min with 50 μM H_2_O_2_ to create an oxidative stress without being toxic to the cells. Cells were then washed, resuspended in PBS, and kept on ice for an immediate detection by flow cytometry (FACSCanto II, Becton Dickinson, San Juan, CA, USA). Data was acquired and analyzed using the FACSDiva software (Becton Dickinson, San Juan, CA, USA). For quantification of DHE, samples were acquired in triplicate and 10,000 events were used for each measurement. Cells were excited at 488 nm and DHE was detected using 585/42 bandpass filter and data expressed as the median fluorescence intensity (MFI).

### Measurement of oxidized DNA by alkaline comet assay

The DNA damage was assessed using alkaline single cell gel electrophoresis (the alkaline comet assay), following established protocols from our laboratory [11,26,35] based on Singh et al. [15] with minor modifications and under low brightness and controlled temperature due to the photo and thermo sensitivity of the assay.

The comet assay is a well validated technique for measurement of DNA damage in individual cells [56]. In brief, histological slides were precoated with 1.5% normal melting point agarose in PBS in a water-bath at 65°C. Subsequently, 20 μL of cell suspension was embedded in 100 μL of 0.5% low melting point agarose in PBS at 37°C and spread on agarose-precoated slides using coverslips. After gelling at 4°C for 20 min, the coverslips were removed and the slides were immersed in freshly prepared lysis solution (2.5 M NaCl, 100 mM EDTA, 10 mM Tris, 34 mM N-Lauroylsarcosine sodium, adjusted to pH 10.0-10.5, using freshly added 1% Triton X-100 and 10% DMSO) for 1 hour at 4°C. After washing in cold distilled water, the slides were quickly immersed in PBS solution. Then, the slides were placed in an electrophoresis chamber filled with freshly prepared alkaline buffer (300 mM NaOH, 1 mM EDTA, pH > 13) for 40 min at 4°C, and electrophoresed at 300 mA and 20 V for 30 min. Afterwards, the slides were neutralized with a 0.4 M Tris buffer (pH 7.5), for 5 min, washed with cold distilled water and allowed to dry at room temperature for 1 hour.

Migration of DNA fragments towards the anode creates a comet ‘tail’, visualized by staining with ethidium bromide (20 μg/mL, Sigma-Aldrich). Immediately afterwards, images were obtained at a magnification of 20x using a fluorescence optical microscope (Nikon Eclipse TI, Melville, NY, USA) equipped with excitation (510-550 nm) and barrier (590 nm) filters. The coded images were acquired using a CCD camera (Nikon) and were analyzed with the CASP program (public domain). Among several parameters provided by the program CASP, we used the percentage of DNA in the tail and the tail moment for analysis of DNA damage. Comets with more than 25% of tail DNA were categorized as moderate-to-high damage and quantified. All samples were coded before scoring. The images of 100 randomly selected cells from each sample obtained from two replicate slides for each animal were analyzed. During the image analysis, comets without clearly identifiable heads or comets with most of their DNA in their tails after electrophoresis were excluded, as a quality control parameter.

### Statistical analysis

All data are expressed as the mean ± SEM. The Kolmogorov-Smirnov test showed that variables had a normal (Gaussian) distribution. The statistical analysis was performed using the one-way analysis of variance (ANOVA). When the ANOVA showed significant differences, the Bonferroni’s test was performed as a *post hoc* analysis. The differences were considered significant when p < 0.05.

## Competing interests

The authors declare no conflict of interest.

## Authors’ contributions

BPR carried out experimental analysis and acquisition of data, analysis and interpretation of the data and drafted the manuscript. BPC participated in the study’s design, supervision in the critical revision of the manuscript and carried out the experimental analysis. CMB carried out the lipid profile analysis, treated the animals and participated in the critical revision of the manuscript. TMCP participated in the supervision and in the critical revision of the manuscript. SSM and ECV contributed to the conception, design and supervision of the study and interpretation of data. All authors read and approved the final version of the manuscript.
